# Formation of Nanostructured Carbon from [Ni(NH_3_)_6_]_3_[Fe(CN)_6_]_2_

**DOI:** 10.3390/nano10020389

**Published:** 2020-02-23

**Authors:** Denis P. Domonov, Sophiya I. Pechenyuk, Alexander T. Belyaevskii, Kirill V. Yusenko

**Affiliations:** 1 Tananaev Institute of Chemistry and Technology of Rare Elements and Mineral Raw Materials of RAS KSC (ICTREMR), Akademgorodok, 26a, Apatity, 184209 Murmansk Region, Russia; s.pecheniuk@ksc.ru (S.I.P.); a.beliaevskii@ksc.ru (A.T.B.); 2 Bundesanstalt für Materialforschung und -prüfung BAM, Richard-Willstätter Str. 11, D-12489 Berlin, Germany

**Keywords:** double complex compound, thermal decomposition, carbon materials

## Abstract

The products of thermal decomposition in an argon atmosphere of [Ni(NH_3_)_6_]_3_[Fe(CN)_6_]_2_ as a precursor has been studied. Decomposition products were studied up to 800 °C. Above 600 °C, all coordination bonds in the residues are broken with a formation of Ni_3_Fe, Fe, and free carbon with a small admixture of nitrogen. Elementary carbon can be easily separated from metals by treatment with a water solution of hydrochloric acid. Only carbon is responsible for the specific surface of the composite products. The released carbon has a high degree of graphitization and begins to oxidize in air above 500 °C and is completely oxidized above 700 °C.

## 1. Introduction

Double complex compounds (DCC) represent a large class of coordination compounds containing coordination cations and coordination anions. Recently, DCC were proposed as promising precursors for the preparation of nanostructured polymetallic alloys [1]. In recent studies, metallic products obtained after thermal decomposition of DCC were also proposed as active heterogeneous catalysts for CO oxidation [2,3]. The thermal decomposition of DCC with 3*d* metals coordinated with carbon-containing ligands results in a formation of large amounts of residual carbon (20–40 wt.% of the initial carbon [4,5,6,7,8],) in an inert and reducing atmosphere. If DCC contains cyano-groups in an anionic part, residual carbon forms easily in an inert atmosphere. The resulting product of thermal decomposition can be considered as a metal/carbon composite material.

Recently, metal/carbon composites based on pure Fe, Co, and Ni, as well as their alloys were proposed as effective materials for converting high-frequency electromagnetic radiation into thermal energy [9,10,11]. So, the thermal decomposition of carbon-containing DCC can be considered as a possible routine to access metal/carbon composites.

Among DCC of the first transition row metals [4,12,13], [Ni(NH_3_)_6_]_3_[Fe(CN)_6_]_2_ [4] has been investigated in many details to understand its thermal behavior in various atmospheres. It has been shown that it forms the highest amount of residual carbon in an inert atmosphere (up to 50% [4,5]). Nevertheless, the process of the formation of residual carbon and its properties have not yet been specifically studied. [Ni(NH_3_)_6_]_3_[Fe(CN)_6_]_2_ seems to be an attractive single-source precursor for the preparation of Fe–Ni–C compositions, since nickel and iron are capable of forming intermetallic compounds, alloys, and spinel type mixed oxides.

It has been shown that the crystal structure of DCC effects their thermal decomposition [5]. However, low stability of [Ni(NH_3_)_6_]_3_[Fe(CN)_6_]_2_ makes the determination of its crystal structure and growing its single crystals difficult. A [Ni(NH_3_)_6_]^2+^ cation undergoes easy hydrolysis in water solution due to its low stability constant (ca. 10^6^). Therefore, the compound can be successfully prepared in the presence of a large excess of ammonia in water solution. In air, a dry compound loses coordinated ammonia and undergoes fast degradation, so it should be stored in closed vessels under a small amount of ammonia pressure. As a result, only powder diffraction data can be applied for its structural characterization.

In air flow, [Ni(NH_3_)_6_]_3_[Fe(CN)_6_]_2_ releases coordinated ammonia in two stages at 125 and 300 °C. Coordinated cyanide ligands decompose in the region of 200–480 °C with the formation of carbon dioxide, nitrogen, and nitrous oxide [4,5,13]. The solid residue is a mixture of nickel and iron oxides.

In hydrogen flow, ammonia also releases in two stages. Additionally, *ca.* 3 mol HCN can be detected. Carbon releases as hydrocarbons, mainly methane, as a product of hydrogenation of cyano-groups to hydrocarbons and ammonia [13]. Above 870 °C, carbon cannot be detected in final solid products; only a mixture of Ni_3_Fe and an *fcc*-structured Fe–Ni alloy can be isolated.

In argon flow, ammonia also releases in two stages at 120 and 400 °C. Between 3 and 3.5 mol of ammonium cyanide releases in a single step at 380 °C. The rest of the carbon remains in the solid phase in the form of cyano-groups, which decompose at 550–600 °C with the release of molecular nitrogen. 1–2 N atoms still remain in the solid residue and can be gradually removed by heating above 1000 °C.

In the current study, we perform a detailed investigation of the formation process and the properties of free carbon formed in the solid residue upon the thermal decomposition of [Ni(NH_3_)_6_]_3_[Fe(CN)_6_]_2_ in an inert atmosphere. The main attention has been paid to understand a process of carbon formation from coordinated cyano-groups.

## 2. Materials and Methods

[Ni(NH_3_)_6_]_3_[Fe(CN)_6_]_2_ was prepared from water solution by mixing equivalent amounts of [Ni(NH_3_)_6_]Cl_2_ (synthesized according to [14]) and K_3_[Fe(CN)_6_]. For C_12_H_54_N_30_Ni_3_Fe_2_, calc. (wt.%): C, 15.9; Ni, 19.4; Fe, 12.3; found (wt.%): C, 15.8; Ni, 19.7; Fe, 12.3. For C_12_H_36_N_24_Ni_3_Fe_2_ prepared from [Ni(NH_3_)_6_](NO_3_)_2_: found (wt.%): C, 17.7; Ni, 21.8; Fe, 14.0; calc. (wt.%): C, 17.9; Ni, 21.9; Fe, 13.9.

**Figure 1:**
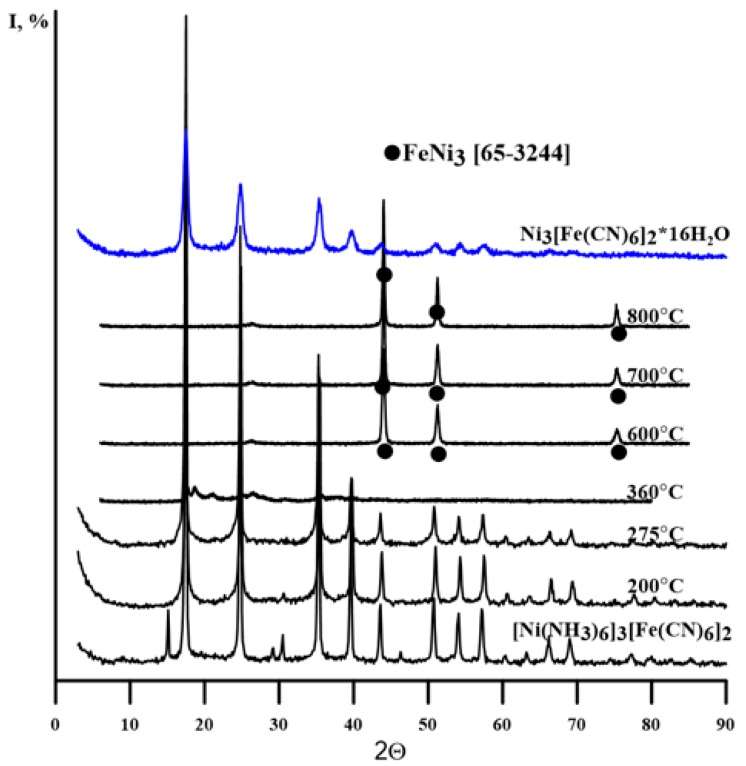
Powder X-ray diffraction (PXRD) patterns of [Ni(NH_3_)_6_]_3_[Fe(CN)_6_]_2_, Ni_3_[Fe(CN)_6_]_2_^.^16H_2_O [4] and its thermolysis products.

**Figure 2:**
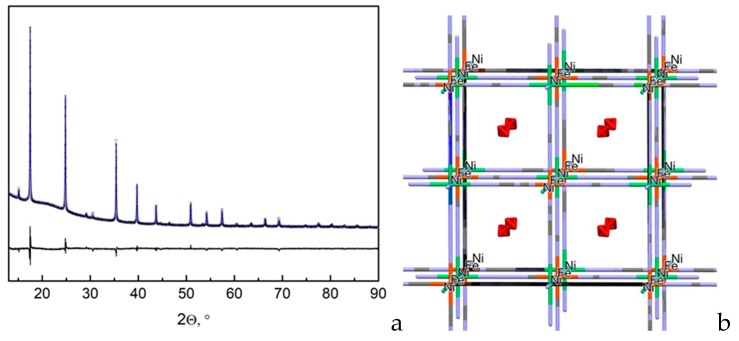
Rietveld refinement corves of the crystal structure of [Ni(NH_3_)_4_]_3_[Fe(CN)_6_]_2_ (dots—experimental diffractogram; solid line—calculated diffractogram; difference curve is shown below) (**a**) (left). The model of the crystal structure of [Ni(NH_3_)_4_]_3_[Fe(CN)_6_]_2_ (disordered ammonia molecules are shown in the middle of the cavities) (**b**).

A carbon elemental analysis was performed on an ELTRA CS-2000 (Alpha Resources, LLC, Stevensville, MI, USA) automated analyser. To determine the metal content, weighed portions of the complex or its thermolysis products were dissolved in a mixture of concentrated HNO_3_ and HCl. The resulting solutions were analyzed by the atomic absorption method on a spectrometer AAnalyst 400 (PerkinElmer, Inc. Waltham, MA, USA). Powder X-ray diffraction (PXRD) patterns of the compound and its thermolysis products (Figure 1) were obtained on a Shimadzu XRD 6000 (Shimadzu Corp., Columbia, MD, USA) diffractometer using Cu*K_α_* radiation (graphite monochromator, λ = 1.54Å) and were compared with data from the Powder Diffraction File (PDF) database [15]. The diffraction pattern of the starting salt was collected in transmission geometry on a BRUKER Advance diffractometer (BRUKER AG, Karlsruhe, Germany, Cu*K_α1_* radiation, Johansson monochromator, the sample was placed in a 0.5 mm glass capillary and sealed in air). The whole-profile Rietveld refinement was performed using Jana2006 [16] (Figure 2).

**Figure 3:**
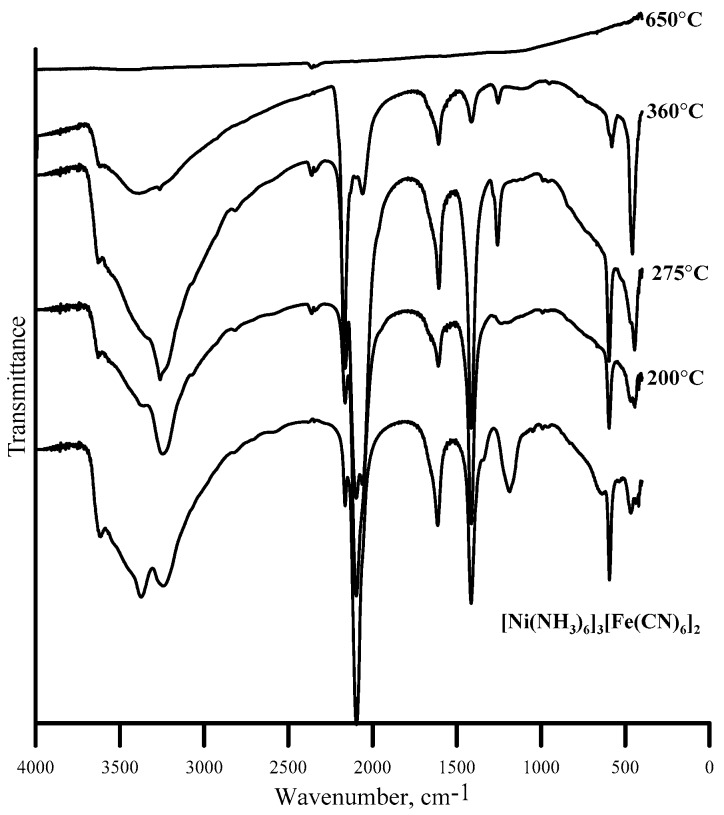
IR spectra of [Ni(NH_3_)_6_]_3_[Fe(CN)_6_]_2_ and its thermolysis products in argon.

The infrared (IR) spectra of the initial complex and solid thermolysis products (Figure 3) were recorded on a Nicolet 6700 FT-IR spectrometer (Thermo Fisher Scientific Inc., Hillsboro, OR, USA) in KBr pellets. The spectra were interpreted based on [17]. The characteristic absorption maxima in IR spectra are summarized in Table 1.

**Figure 4:**
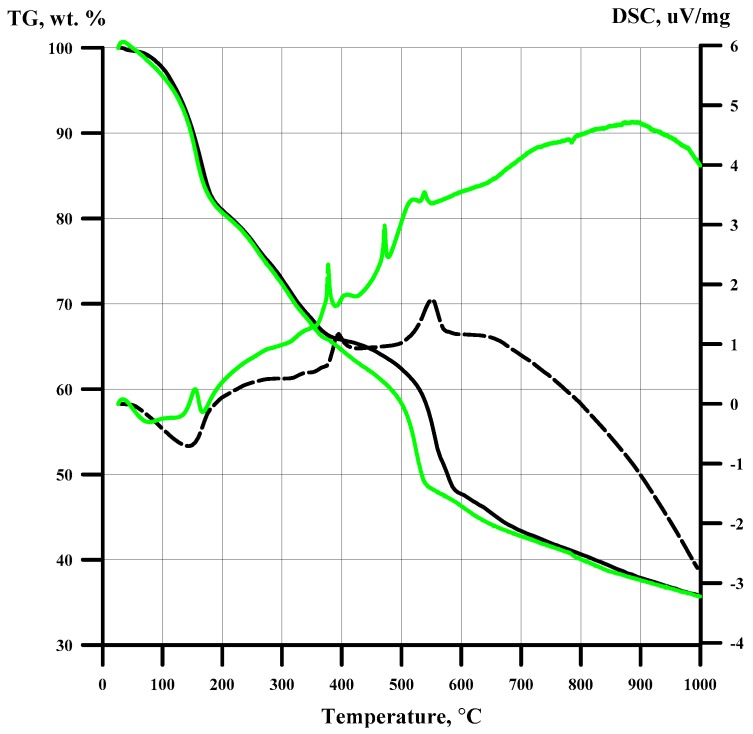
TG and DSC curves for [Ni(NH_3_)_6_]_3_[Fe(CN)_6_]_2_ (10 (─) and 5 (**─**)°/min).

The specific surface area was measured on a Tristar 3020 and FlowSorbII 2300 from Micromeritics (Micromeritics Instrument Corp. Norcross, GA, USA). Thermal analysis study (Differential thermal analysis, DTA and Thermogravimetric analysis, TG) was carried out in an argon flow on a NETZSCH STA 409 PC/PG instrument (NETZSCH-Gerätebau GmbH, Selb, Germany) in a corundum crucible with a lid (sample weights 7–10 mg, gas flow rate 40 ml/min, heating rate 5 and 10 K/min, temperature range 20–1000 °C). Thermal analysis curves are presented in Figure 4.

In order to isolate thermolysis products, weighed portions of the compound were heated at a rate of 5 °C/min to a predetermined temperature and were then kept at this temperature for 1 h. The samples were placed in quartz vessels and heated in a NaberthermRT 50-250/11 programmable tube furnace (Nabertherm GmbH, Lilienthal, Germany) under argon flow. The products were cooled in an argon atmosphere for 24 h. For the determination of evolved gases, gaseous products were passed through a vessel with Raschig rings cooled by ice and were then passed through two sequential Drexel flasks with an HCl solution and a mixture of NaOH and H_2_O_2_ solutions. The amount of ammonia was analysed in acidic solution to obtain the amount of evolved NH_3_. In alkaline solution, nitrate ions were analysed to obtain amount of evolved HCN.

Carbon was recovered from calcination residues by leaching with cold or hot 6M HCl. Insoluble carbon was filtered on Schott glass filters No. 3, washed with boiling distilled water, and dried at 100 °C to constant weight. Carbon prepared by the heating of glucose powder under the same conditions (argon flow in a flow reactor at 700 °C) has been used for comparison.

Scanning electron microphotographs were obtained using a SEM Leo 420 instrument (LEO, Assing, Italy).

## 3. Results and Discussion

Nickel(II) weakly coordinates ammonia (see above); therefore, the amount of coordinated ammonia in the inner sphere of nickel(II) species might depend on the nature of the initial nickel salt. The reaction of Ni(II) [Fe(CN)_6_]^3-^ anions ammonia water solution results in the quick precipitation of double complex salt with a total composition close to [Ni(NH_3_)_6_]_3_[Fe(CN)_6_]_2_. Its powder X-ray diffraction (PXRD) pattern can be indexed in the cubic space group *Fm*$\bar{3}$*m* with *a* = 10.139(3) Å (Figure 2). [Ni(NH_3_)_6_]_3_[Fe(CN)_6_]_2_ is isostructural to classical Prussian blue compounds, such as Zn_3_[Fe(CN)_6_]_2_∙*n*H_2_O [18], as well as to previously studied Ni_3_[Fe(CN)_2_∙16H_2_O [4] (Figure 1). In Prussian blue analogous, [Fe(CN)_6_]^3-^ ions occupy corners and centres of the faces in the cubic cell with 2/3 occupancy; Ni^2+^ cations occupy the middle of the edges and the centre of the unit cell. Crystal water is disordered over several positions and appears in the centres of the cages. Similarly, the structure of [Ni(NH_3_)_4_]_3_[Fe(CN)_6_]_2_ can be described as a framework formed by [Fe(CN)_6_]^3-^ anions with Ni^2 +^ ions interconnected by Ni^2+^–N–C–Fe^3+^ bridges with isolated [Ni(NH_3_)_6_]^2+^ ions surrounded by disordered ammonia molecules. In the ideal case, Ni^2+^ and [Ni(NH_3_)_6_]^2+^ cations should have 2:1 ratio; so, 2/3 of the nickel ions are linked by cyanide bridges to hexacyanide ions, and 1/3 are isolated [Ni(NH_3_)_6_]^2+^. The ammonia molecules should be in the cages of the structure, since the synthesis is carried out from concentrated ammonia solutions. The IR spectra of [Ni(NH_3_)_6_]_3_[Fe(CN)_6_]_2_ do not show the presence of crystal water in the structure (Table 1).

According to the IR spectra, intermediate products obtained at 200, 275, and 360 °C contain ammonia as well as cyanides coordinated to Fe and Ni. Nevertheless, the absorption intensities are steadily decreasing due to the decreasing of number of coordinated groups (Table 2). At 600–650 °C, coordinated ligands cannot be detected (Figure 3). From the beginning of the curve to 170 °C, there is a mass loss of 19.7 wt.%. Up to 370 °C TG curves coincide with rates of 5 and 10 °C/ min. The weight loss is 34%. A long flat slope on a TG curve up to 410 °C gives a weight loss of 14.7 wt.% and most likely corresponds to ammonia release. A relatively steep decline in TG to 590 (green line) and 535 °C (black line) gives a weight loss of 21 and 18.5 wt.%. Residues from calcination at 1000 °C in both cases correspond to 36 wt.% (Figure 4). Ammonia is gradually removed from the sample up to 360 °C (Table 2), and with a further increase in the thermolysis temperature, the amount of ammonia released stabilizes at the level of 6–7 mol/mol of the complex. As it has been noted in early report [4], it is possible to capture only 1/3 of coordinated ammonia. It can be explained due to the catalytic decomposition of gaseous ammonia in the presence of iron cyanides and metallic nickel [4].

The XRD patterns (Figure 1) of the initial compound and the thermolysis products at 200 and 275 °C are almost identical. PXRD patterns can be attributed to a mixture of Ni_2_[Fe(CN)_6_] and Fe_4_[Fe(CN)_6_]_3_ [15]. The structure of such a mixture should not differ too much from the one described above, since nothing prevents the [Fe(CN)_6_]^3-^ anions and nickel cations to occupy above-mentioned crystallographic positions. However, the IR spectra of thermolysis products below 400 °C indicate cyano-groups and ammonia that coordinated as well as NH_4_^+^. At the same time, bands at 2163, 2097, and 2055 cm^−1^ detected in the IR spectra of thermolysis products below 400 °C can be attributed to Ni–NC–Fe^II,III^ bonds, which indicates the presence of nickel not surrounded by coordinated ammonia molecules and confirms the structure shown in Figure 2.

A comparison of the previously obtained [4,5] and new data allows us to conclude that in the range 200–400 °C the products of thermolysis contain from 15 to 2–3 ammonia molecules. At higher temperatures, carbon and nitrogen are retained in the thermolysis product and cannot be indicated in the IR spectra. Previously [4,5], it was shown that ammonium cyanide (hydrogen cyanide) can be detected upon thermal decomposition in an inert flow of [Ni(NH_3_)_4_]_3_[Fe(CN)_6_]_2_ (at 310–420 °C). The dissociation of cyano-groups to carbon and nitrogen occurs at 600–650 °C. The PXRD patterns of thermolysis products above 650 °C show only Ni_3_Fe and Fe reflections.

**Figure 5:**
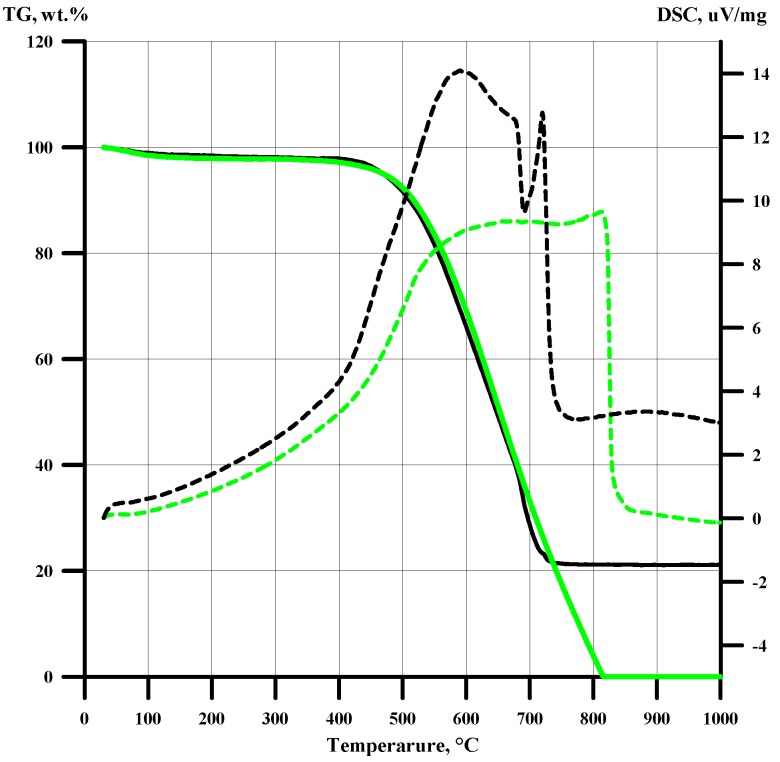
Thermogravimetry (solid lines) and differential thermal analysis (dashed lines) curves of carbon obtained from [Ni(NH_3_)_6_]_3_[Fe(CN)_6_]_2_ in argon at 700 °C (─) and from glucose at 900 °C(**─**).

**Figure 6:**
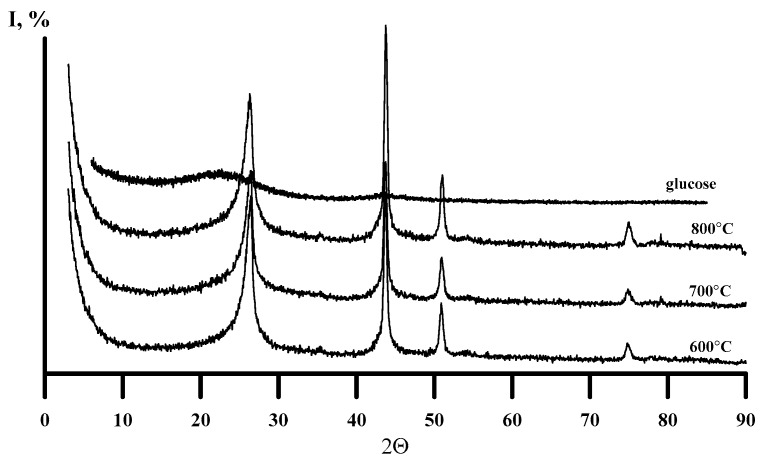
PXRD patterns of carbon samples isolated from thermolysis products and glucose.

**Figure 7:**
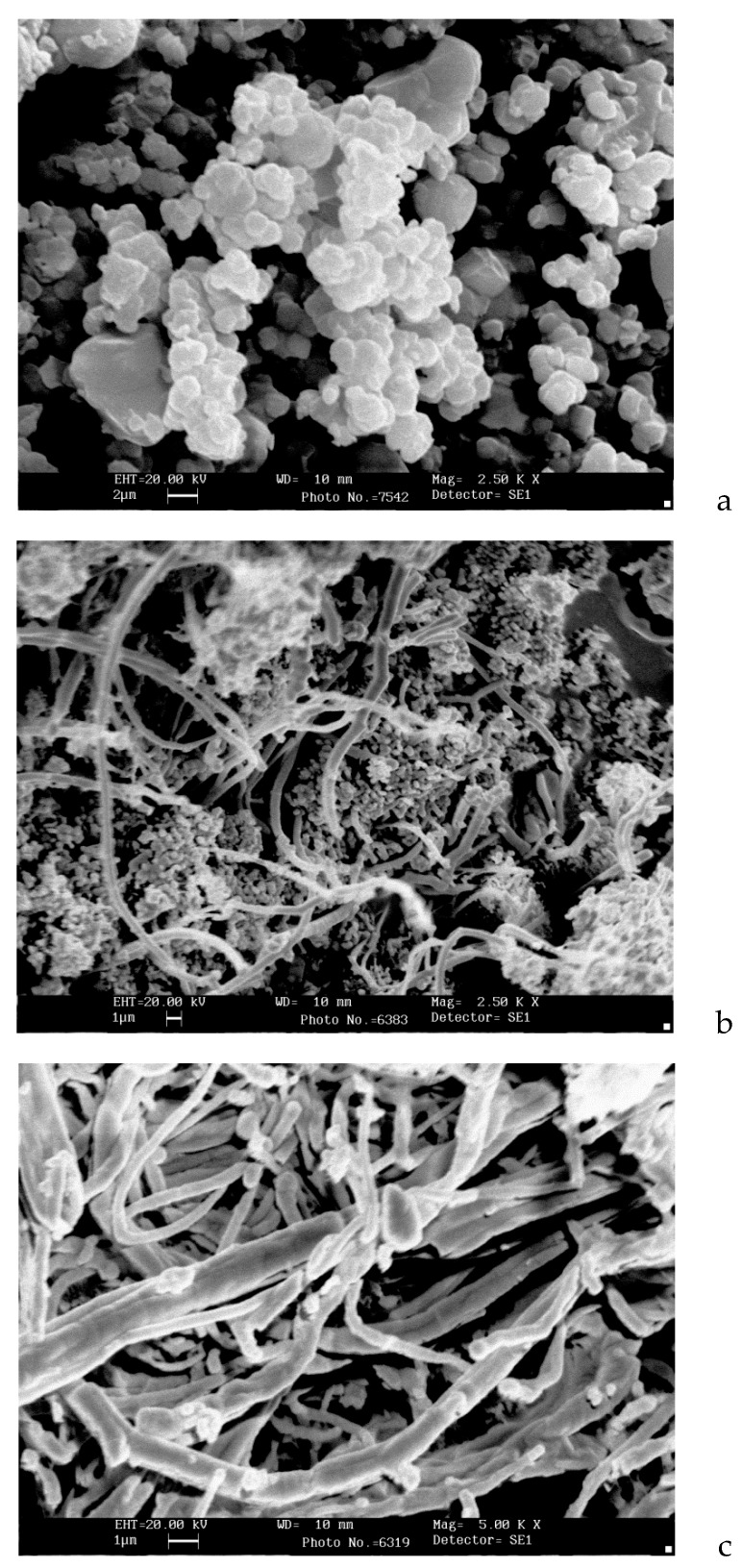
Micrographs of [Ni(NH_3_)_6_]_3_[Fe(CN)_6_]_2_ (**a**), its thermolysis product at 650 °C (**b**), and carbon (**c**) isolated from the thermolysis product at 650 °C.

It is possible to isolate the residual carbon from the products of thermolysis of DCC in an argon atmosphere obtained above 600 °C. Thermolysis product obtained at 360 °C was dissolved in HCl. After dissolution, ferrocyanide ions were detected in solution. Similar dissolution was performed for products obtained at 600, 650, 700, and 800 °C (Figure 5, Figure 6 and Figure 7). So, metallic particles can be easily dissolved from the composite in a concentrated HCl water solution. The amount of carbon obtained from the thermolysis products quite closely corresponds to the analytically determined carbon content and might exceed by 10–30 wt.%. This carbon contains small Ni and Fe admixtures (Table 3).

Figure 5 shows the curves of thermal analysis in the air of the obtained carbon samples. For comparison, Figure 5 and Figure 6 show the characteristics of the glucose thermolysis product at 900 °C in argon. Carbons extracted after thermal decomposition of DCC are similar to thermally expanded graphite [19,20]. All samples have high surface areas and are stable in air below 450 °C. In [21], the high-temperature graphitization of carbon materials obtained as products of pyrolysis of the initial fibrous precursor poly (p-phenylenebenzo-bis-oxazole (PBO) were described. It has been found, that even at 2400 °C, the interlayer distance *d*_002_ = 3.42 Å in graphitic carbon obtained from PBO still exceeds the characteristic value for pure crystalline graphite (3.354 Å). Figure 6 shows XRD patterns of carbon samples that were extracted from products of the thermal decomposition of [Ni(NH_3_)_4_]_3_[Fe(CN)_6_]_2_ at 600–800 °C. Corresponding diffraction (0 0 2) peak positions (2θ = *ca*. 26°) and interlayer distances *d*_002_ (Figure 6, Table 3) are comparable with the relevant data for PBO graphitization products at 2400 °C [21]. Carbon obtained from glucose (absence of metal in the initial material) shows no visible crystallinity. It can be an indication for catalytic role of metals from DCC in the low-temperature graphitisation process [22].

The total specific surface area characteristic for obtained carbon powders is similar to the total surface area of composites before the dissolution of metals. For example, the residue from the calcination of the complex in argon flow at 650 °C has a specific surface area of 140.5 m^2^/g and a residual carbon content of 23.9 wt.%. From 3.0 g of the residue, 0.82 g of carbon with a specific surface of 148 m^2^/g was obtained. The total surface of 3.0 g of the residue is 121.5 m^2^; the surface of 0.82 g carbon obtained after dissolution of metals is 121.4 m^2^/g (Table 3). The ratio between the *S_sp_* of residues from calcination and the carbon released from them suggests that, in the residue from calcination, carbon envelops metal particles, providing the entire surface of the residue from calcination.

Figure 7a shows that the initial complex is crystallized in the form of hexagonal plates with a size of 1.5–2 microns. The thermolysis product (Figure 7b) has a loose fibrous structure with a fibre thickness of 0.5–1 μm. Almost the same structure is preserved for the released carbon (Figure 7c). The diameter of the fibre is 1–2 μm.

## 4. Conclusions

[Ni(NH_3_)_6_]_3_[Fe(CN)_6_]_2_ obtained from concentrated ammonia solutions has a complex crystal structure. Its cubic lattice is formed by [Fe(CN)_6_]^3-^ ions occupying the tops and centres of the faces, where the Ni^2+^ and [Ni(NH_3_)_6_]^2+^ occupy the midpoints of the edges and the centres of the cell. The voids in the crystal structure are probably occupied by ammonia molecules. Upon thermal decomposition in an inert atmosphere, NH_3_ ligands can be removed in two stages. At the first stage (below 300 °C), ammonia can be removed from the voids. Further heating results in coordinated ammonia evolving. Nevertheless, at 300 °C, coordinated ammonia molecules can still be obtained in samples. Above 360 °C, only traces of ammonia can be detected. Above 600 °C, [Ni(NH_3_)_6_]_3_[Fe(CN)_6_]_2_ completely degrades with a formation of Ni_3_Fe, Fe, and carbon with a small nitrogen admixture. Since carbon was present only in the anion, it is undeniable that it is a decomposition product of coordinated cyano-groups. Metals can be easily leached from residues by acid treatment. Residual carbons show a significant degree of graphitization. The ratio between the S_sp_ of residues from calcination and the carbon released from them suggests that, in the residue from calcination, carbon envelops metal particles, providing the entire surface of the residue.

## Figures and Tables

**Table 1:** Characteristic absorption lines in the IR spectra of thermolysis products.

Thermolysis Temperature, °C	Absorption Lines, cm^−1^
starting [Ni(NH_3_)_4_]_3_[Fe(CN)_6_]_2_	3374, 3242 ν(NH_3_); 2169, 2095 ν(C≅N); 1613 δ_d_(NH_3_); 1415 δ_d_(NH_4_); 1186 δ_s_(NH_3_); 594 ρ_r_ (NH_3_); 467 ν(M-N) ^1^
200	3367, 3244 ν(NH_3_); 2163, 2097 ν(C≅N); 1610 δ_d_(NH_3_); 1414 δ_d_(NH_4_); 596 ρ_r_(NH_3_); 444 ν(M-N)
275	3261 ν(NH_3_); 2162, 2097, 2055 ν(C≅N); 1607δ_d_(NH_3_); 1414 δ_d_(NH_4_); 1258 δ_s_(NH_3_); 596 ρ_r_(NH_3_); 445 ν(M-N)
360	3386, 3156 ν(NH_3_); 2167, 2059 ν(C≅N); 1608 δ_d_(NH_3_); 1413 δ_d_(NH_4_); 1254 δ_s_(NH_3_); 580 ρ_r_(NH_3_); 457 ν(Fe-N)

**^1^** The bands at *v* <500 cm^−1^ can be caused by stretching vibrations of both Ni—N and Fe—C bonds. [17].

**Table 2:** The results of the experiments on the thermolysis of [Ni(NH_3_)_6_]_3_[Fe(CN)_6_]_2_.

T^1^, °C	Residue, wt.%	M.m.^2^	Composition, wt.%			Released Ammonia, mol	S_sp_^3^, m^2^/g	Description of Samples
			Ni	Fe	C			
starting	100	905.7 803.7	19.44 21.8	12.3 14.0	15.9/100 17.7/100	– –	–	Ni_3_Fe_2_C_12_H_54_N_30_ = [Ni(NH_3_)_6_]_3_[Fe(CN)_6_]_2_ Ni_3_Fe_2_C_12_H_36_N_24_ = [Ni(NH_3_)_4_]_3_[Fe(CN)_6_]_2_
200	83.9 79	736 660	23.9 26.3	15.1 17.1	19.7 20.9	– 2.7	–	Ni_3_Fe_2_C_12_H_24_N_20_ = [Ni_3_(NH_3_)_8_][Fe_2_(CN)_12_][4] Ni_3_Fe_2_C_11.5_H_12_N_15.5_ = [Ni_3_(NH_3_)_4_][Fe_2_(CN)_11.5_]
275	79 74	736 630	23.9 28.1	15.0 18.0	19.1 21.2	– 4.4	– –	Ni_3_Fe_2_C_12_H_24_N_20_ = [Ni_3_(NH_3_)_8_][Fe(CN)_6_]_2_ Ni_3_Fe_2_C_11.5_H_9_N_15.5_ = [Ni_3_(NH_3_)_3_][Fe_2_(CN)_11_]
360	66.3	600	29.7 31.4	18.2 18.7	20.4/85.1	5.0	170	Green residue Ni_3_Fe_2_(CN)_10.4_
600	41.08	372	47.54	29.56	23.2	–	27.71	Ni_3_Fe_2_C_7.3_
650	44.3	401 (375)	–	–	23.9/66.6	6.8	40.5	Black, loose, Ni_3_Fe_2_C_7_N_2_ Sharp lines of Ni_3_Fe
650	44.4	402 (375)	49.0	29.8	22.0/61.4	6.5	43.8	
700	39.9	361	48.80	31.54	18.7	–	72.73	Ni_3_Fe_2_C_5.5_N_0.5_
800	45 41.6	400 377	44.4 48.43	28.5 31.23	21.6 23,6	6.2 –	78,54 77.52	Ni_3_Fe_2_C_7_N_2_ Ni_3_Fe_2_C_7_N_0.5_
1000	46.2	400	44.4	28.1	21.0	–	–	Ni_3_Fe_2_C_7_ [4]

^1^ Thermolysis temperature. ^2^ Molecular mass. ^3^ S_sp_ is the surface area of thermolysis product.

**Table 3:** The yield and properties of carbon from the product thermolysis of [Ni(NH_3_)_6_]_3_[Fe(CN)_6_]_2._

Thermolysis Temperature, °C	Carbon Yield, g/g	S_sp_, m^2^/g	*d_002_*_, Å_	Crystallite Size, nm	Content of Metals in Carbon, wt.%	
					Ni	Fe
**600**	**0.30**	**224**	**0.341**	14	–	–
650	0.27 0.24	148 226	0.337 0.335	36 38	4.2 –	3.4 –
700	0.28	276	0.341	14	8.03	6.77
800	0.33 0.30	230 209	0.343 0.341	40 42	10.4 8.44	7.5 6.12

## References

[1] Korenev S.V., Venediktov A.B., Shubin Y.V., Gromilov S.A., Yusenko K.V. (2003). Synthesis and Structure of Binary Complexes of Platinum Group Metals - Precursors of Metallic Materials. J. Struct. Chem..

[2] Shubin Y.V., Korenev S.V. (2010). Formation of Nanosized Bimetallic Particles Based on Noble Metals. Catal. Ind..

[3] Zadesenets A.V., Garkul I.A., Filatov E.Y., Plyusnin P.E., Filippov T.N., Asanova T.I., Korolkov I.V., Baidina I.A., Asanov I.P., Korenev S.V. (2019). Oxalato complexes of Pd(II) with Co(II) and Ni(II) as single-source precursors for bimetallic nanoalloys. J. Therm. Anal. Calorim..

[4] Pechenyuk S.I., Domonov D.P., Shimkin A.A., Ivanov Y.V. (2015). Thermal decomposition of iron cyano-complexes in an inert atmosphere. Russ. Chem. Bull..

[5] Pechenyuk S.I., Domonov D.P., Shimkin A.A., Semushina Y.P., Ivanov Y.V. (2017). Thermal behavior of binary complex compounds containing the hexacyanoferrate anion. Russ. J. Gen. Chem..

[6] Ng C.W., Ding J., Shi Y., Gan L.M. (2001). Structure and magnetic properties of copper(II) hexacyanoferrate(III) copounds. J. Phys. and Chem. Solids.

[7] Ng C.W., Ding J., Wang L., Gan L.M., Quek C.H. (2000). Thermal-Induced Microstructural Changes of Nickel-Iron Cyanide. J. Pys. Chem. A.

[8] Ng C.W., Ding J., Gan L.M. (2001). Microstructural Changes Induced by Thermal Treatment of Cobalt(II) Hexacyanoferrate (III) Compound. J. Solid State Chem..

[9] Song Z., Liu X., Sun X., Li Y., Nie X., Tang W., Yu R., Shui J. (2019). Alginate-templated synthesis of CoFe/carbon fiber composite and the effect of hierarchically porous structure on electromagnetic wave absorption performance. Carbon.

[10] Ye F., Song Q., Zhang Z.C., Li W., Zhang S.Y., Yin X.W., Zhou Y., Tao H., Liu Y., Cheng L. (2018). Direct growth of edge-rich graphene with tunable dielectric properties in porous Si3N4 ceramic for broadband high-performance microwave absorption. Adv. Funct. Mater..

[11] Shahzad F., Alhabeb M., Hatter C.B., Anasori B., Man Hong S., Koo C.M., Gogotsi Y. (2016). Electromagnetic interference shielding with 2D transition metal carbides (MXenes). Science.

[12] Pechenyuk S.I., Domonov D.P., Rogachev D.L., Belyaevskii A.T. (2007). Anion effect on the thermolysis of double complexes [Co(NH_3_)_6_][Fe(CN)_6_] and [Co(NH_3_)_6_]_4_[Fe(CN)_6_]_3_. Russ. J. Inorg. Chem..

[13] Pechenyuk S.I., Domonov D.P., Avedisyan A.A., Ikorskii S.V. (2010). Conversions of coordinated ligands by reducing thermolysis of some double complex compounds. Russ. Inorg. Chem..

[14] Brauer G. (1978). Handbuch der Präparativen Anorganischen Chemie: In Drei Bänden.

[15] JCPDS-JCDD Card (2002). Newtown Square (PA, USA): International Centre for Diffraction Data.

[16] Petříček V., Dušek M., Palatinus L. (2014). Crystallographic computing system JANA2006: General Features. Z. Kristallogr..

[17] Nakamoto K. (2009). Infrared and Raman Spectra of Inorganic and Coordination Compounds.

[18] Gravereau P., Garnier E. (1984). Structure de la phase cubique de l’hexacyanoferrate(III) de zinc: Zn_3_[Fe(CN)_6_]_2_.nH_2_O. Acta Crystallogr. Sect. C.

[19] Makhorin K.E., Zayats N.N., Donchak S.S. (1990). Analiz derivatogramm okislennogo i vspuchennogo grafita. Khim. tekhnologiya..

[20] Kalashnikova M.Y. (2001). Derivatographic study of thermally expanded graphite products. Vestnik PGTU. Problems of modern materials and technologies. Permian.

[21] Vazquez-Santos M.B., Geissler E., Laszlo K., Rouzaud J.N., Martinez-Alonso A., Tascon J.M.D. (2012). Comparative XRD, Raman and TEM Study on graphition of PBO-derived Carbon Fibers. J. Phys. Chem. C.

[22] Domrachev G.A., Lazarev A.I., Kaverin B.S., Egorochkin A.N., Ob”edkov A.M., Domracheva E.G., Markin G.V., Huipe Nava E., Sorokin A.A., Suvorova O.N. (2004). The Role of Carbon and Metal in Self-Assembly of the Iron–Carbon System at Various Component Ratios. Phys. Solid State..

